# Investigating potential biomarkers of acute pancreatitis in patients with a BMI>30 using Mendelian randomization and transcriptomic analysis

**DOI:** 10.1186/s12944-024-02102-3

**Published:** 2024-04-22

**Authors:** Hua Ji, Zheng Tang, Kexin Jiang, Shuang Lyu, Yiwen Zhao, Jiajie Feng, Ruiwu Dai, Hongyin Liang

**Affiliations:** 1 Department of Hepatobilialy Surgery, General Surgery Center, General Hospital of Western Theater Command, Chengdu, 610083 China; 2 Department of General Surgery, Affiliated Hospital of Southwest Medical University, Luzhou, 646000 China; 3 College of Medicine, Affiliated Hospital of Southwest Jiaotong University, College of Medicine, Southwest Jiaotong University, Chengdu, 610031 China

**Keywords:** AP, BMI, Lipid metabolism, Machine learning

## Abstract

**Background:**

Acute pancreatitis (AP) has become a significant global health concern, and a high body mass index (BMI) has been identified as a key risk factor exacerbating this condition. Within this context, lipid metabolism assumes a critical role. The complex relationship between elevated BMI and AP, mediated by lipid metabolism, markedly increases the risk of complications and mortality. This study aimed to accurately define the correlation between BMI and AP, incorporating a comprehensive analysis of the interactions between individuals with high BMI and AP.

**Methods:**

Mendelian randomization (MR) analysis was first applied to determine the causal relationship between BMI and the risk of AP. Subsequently, three microarray datasets were obtained from the GEO database. This was followed by an analysis of differentially expressed genes and the application of weighted gene coexpression network analysis (WGCNA) to identify key modular genes associated with AP and elevated BMI. Functional enrichment analysis was then performed to shed light on disease pathogenesis. To identify the most informative genes, machine learning algorithms, including Random Forest (RF), Support Vector Machine-Recursive Feature Elimination (SVM-RFE), and Least Absolute Shrinkage and Selection Operator (LASSO), were employed. Subsequent analysis focused on the colocalization of the Quantitative Trait Loci (eQTL) data associated with the selected genes and Genome-Wide Association Studies (GWAS) data related to the disease. Preliminary verification of gene expression trends was conducted using external GEO datasets. Ultimately, the diagnostic potential of these genes was further confirmed through the development of an AP model in mice with a high BMI.

**Results:**

A total of 21 intersecting genes related to BMI>30, AP, and lipid metabolism were identified from the datasets. These genes were primarily enriched in pathways related to cytosolic DNA sensing, cytokine‒cytokine receptor interactions, and various immune and inflammatory responses. Next, three machine learning techniques were utilized to identify *HADH* as the most prevalent diagnostic gene. Colocalization analysis revealed that *HADH* significantly influenced the risk factors associated with BMI and AP. Furthermore, the trend in *HADH* expression within the external validation dataset aligned with the trend in the experimental data, thus providing a preliminary validation of the experimental findings.The changes in its expression were further validated using external datasets and quantitative real-time polymerase chain reaction (qPCR).

**Conclusion:**

This study systematically identified *HADH* as a potential lipid metabolism-grounded biomarker for AP in patients with a BMI>30.

**Supplementary Information:**

The online version contains supplementary material available at 10.1186/s12944-024-02102-3.

## Introduction

Acute pancreatitis (AP) triggers a significant inflammatory response caused by a wide range of factors. This sequence of events leads to the activation of pancreatic enzymes within the pancreas, resulting in autodigestion, tissue swelling, bleeding, and potentially, pancreatic tissue necrosis. Key triggers include cholelithiasis and alcohol consumption. Statistical evidence underscores the substantial public health impact of AP, with an incidence rate of 33.74 cases per 100,000 person-years [1]. In certain countries, the incidence rate of AP has risen to 72 cases per 100,000 person-years [2]. Remarkably, the overall mortality rate associated with this variant of pancreatitis is 15%. When organ failure becomes a prominent feature of the disease, the mortality rate surges to a staggering 35% [3]. In Sweden, the annual financial burden of AP is 38,500,000 euros, with the average treatment cost per patient reaching approximately 10,000 euros [4]. The evaluation of the disease's impact underscores the critical need for proactive management strategies and heightened awareness among healthcare providers and the public. Furthermore, this study highlights the essential need for comprehensive and prompt diagnostic and therapeutic interventions. However, the complex pathogenesis of AP at the microlevel presents a formidable challenge, with many aspects yet to be uncovered.

The World Health Organization defined a Body Mass Index (BMI) of 30 or higher as indicating obesity. Obesity substantially affects the development and progression of AP, exerting multiple adverse effects on this condition. Research indicates that obesity can exacerbate the inflammatory response associated with AP, leading to detrimental outcomes [5, 6]. A study conducted in 2014 corroborated these findings, highlighting the strong link between obesity and the exacerbation of the systemic inflammatory response in acute pancreatitis [7]. Individuals with a BMI of 23 or higher face a significantly increased risk of developing severe acute pancreatitis compared to those with a normal BMI [8]. Obesity is acknowledged as a major risk factor for AP, introducing additional complexity to the disease etiology. Considering the established link between obesity and AP, unraveling the potential molecular mechanisms that connect these two conditions is crucial. Gaining such insights is essential for enhancing our understanding of AP pathogenesis and could pave the way for innovative therapeutic approaches for individuals affected by this condition. Furthermore, lipid metabolism has been identified as a key element in the pathogenesis of various diseases, including obesity, AP, cancer, immune disorders, and neurodegenerative diseases [9–12]. The complex interplay between lipid metabolism and disease pathogenesis highlights its importance in understanding disease mechanisms and developing personalized treatment strategies.

Bioinformatics offers a systematic approach to deciphering complex biological processes, aiding in the identification of molecular signatures that underpin disease pathophysiology. Machine learning, a subset of artificial intelligence, bolsters bioinformatics through the use of algorithms capable of identifying patterns and relationships within vast datasets. The integration of machine learning into modern precision medicine is attributed to its ability to accurately process and manage large quantities of data, enhancing the development and application of personalized treatment strategies [13]. By integrating the capabilities of bioinformatics and machine learning, it becomes possible to efficiently explore the complex network of molecular interactions and pinpoint potential biomarkers with clinical significance.

Despite the limited research exploring the causal relationship between BMI and AP and the scarcity of studies identifying shared diagnostic biomarkers for high BMI status and AP, this study sought to address these gaps. Initially, hypothesizing a distinct causal link between BMI and AP complicated by genetic factors, this investigation represents a pioneering effort to combine Mendelian randomization (MR), bioinformatics analysis, and machine learning algorithms to examine the BMI-AP connection. Moreover, this study aimed to identify key genes implicated in the progression of AP in individuals with a BMI >30.

## Methods

### Data collection

#### Exposure

BMI data were collected from the IEU database, specifically from the IEU OpenGWAS project (mrcieu.ac.uk), including samples ukb-a-248, ukb-b-19953, and ukb-b-2303.

#### Outcome

AP data were collected from the IEU database, specifically from the IEU OpenGWAS project, including sample ukb-b-19388.

#### Transcriptomic data

Three RNA sequencing datasets were obtained from the GEO public database (http://www.ncbi.nlm.nih.gov/geo) [14]. These include:

- GSE151839: Gene expression data from skin and fat biopsies of 10 obese (BMI 35-50) and 10 nonobese (BMI 18.5-26.9) individuals.
- GSE44000: Gene expression data from subcutaneous adipose tissue of 7 obese (BMI>30) and 7 nonobese (BMI<25) individuals.
- GSE194331: Gene expression data from whole blood samples were collected from 32 healthy individuals and 87 individuals diagnosed with AP.

**Fig. 1:**
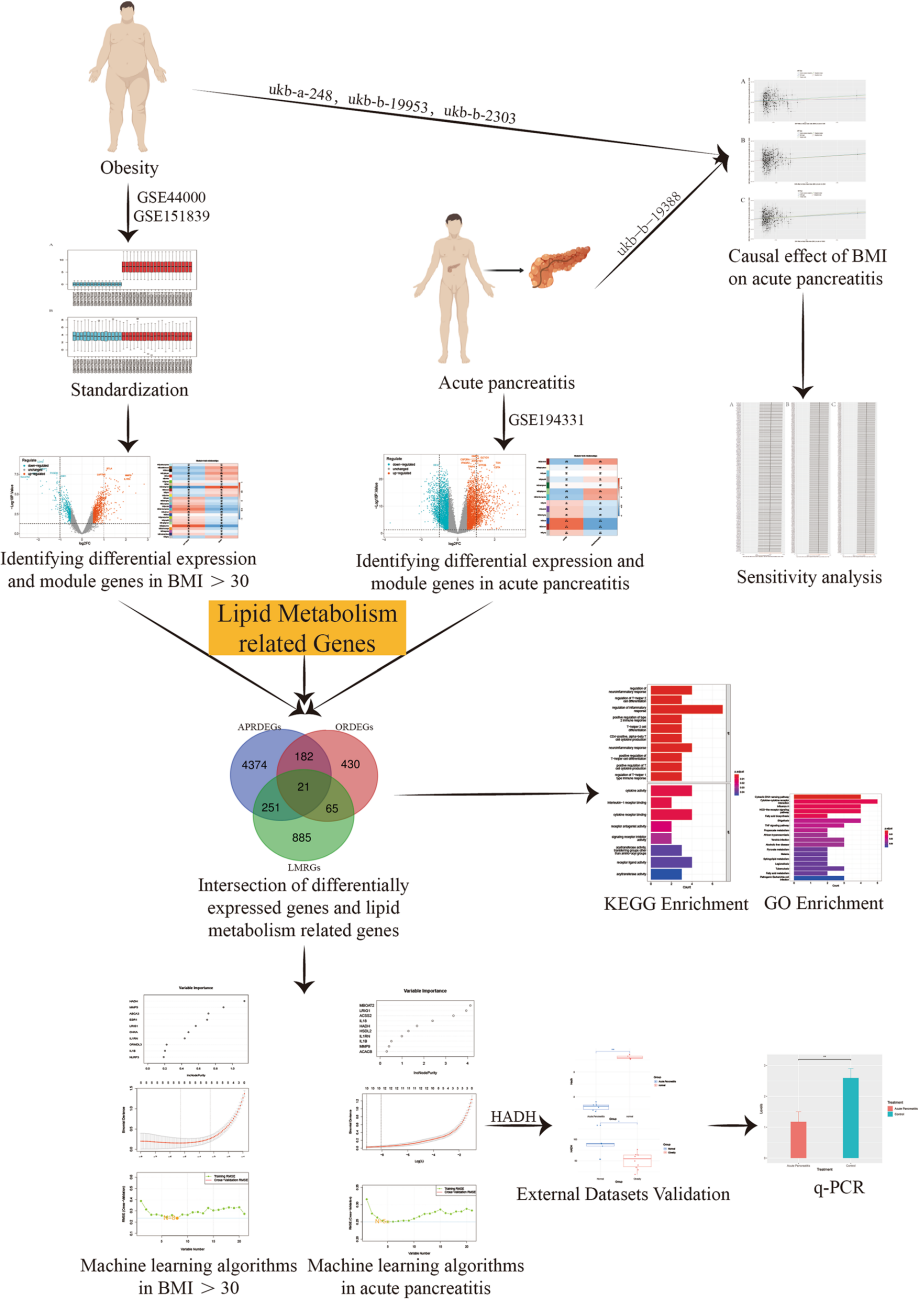
The flow chart of this study

Additionally, 1222 lipid metabolism-related genes (LMRGs) were downloaded from NCBI (National Center for Biotechnology Information (nih.gov), accessed in July 2023) using the keywords "lipid metabolism" and "*Homo sapiens*". The GSE109227 and GSE166047 datasets were used as validation cohorts. The research design is illustrated in the flowchart in Fig. 1.

### Causal effect of BMI on AP

To address linkage disequilibrium, this study excluded SNPs with a r^2^ greater than 0.001 within a 10,000 kb radius of the most significantly associated SNP across three distinct BMI datasets. A significance threshold of *P* < 5×10^−8^ was set, and SNPs meeting this criterion were selected as instrumental variables (IVs) to investigate the causal association between BMI and AP. This study utilized dual-sample MR analysis to determine the magnitude and direction of the impact of BMI on AP. Various MR methods (MR‒Egger, weighted mode, simple mode, inverse variance weighted (IVW) and weighted median) were employed to establish causality, with a preference for the IVW approach due to its robustness. After establishing causality, heterogeneity was assessed to ensure the reliability and consistency of the findings.

### Identification of differentially expressed genes (DEGs)

Fat biopsy gene expression data from GSE151839 were combined with GSE44000 gene expression data. Acknowledging the difficulty of directly comparing samples from different batches and the potential pitfalls of merging datasets without accounting for batch effects and variations, this study addressed this issue using the “sva” R package. Batch effects were removed by preserving only common genes in the merged dataset, facilitating the integration of datasets from different platforms. Outlier samples were excluded through correlation analysis, resulting in 16 samples with BMI>30 and 11 samples with BMI<30 being retained. For GSE194331, after the data were downloaded, genes with an average expression level greater than 1 were retained to increase the reliability of the data. Logarithmic processing was performed on the data, and outlier samples were excluded through correlation analysis, leaving 70 AP samples and 20 control samples. DEGs were identified utilizing the “limma” package (*P* <0.05 and |log2FC|≥0.5) [15]. DEGs were visualized using volcano plots.

### Weighted gene coexpression network analysis (WGCNA)

To elucidate the associations between gene expression levels and diseases, the “WGCNA” package was used to construct a coexpression network. Data preprocessing began with the “goodSamplesGenes” function within the “WGCNA” R package, which effectively removed statistically significant outlier samples. Subsequently, an appropriate soft power parameter (β) was carefully chosen to construct a weighted adjacency matrix, which was subsequently transformed into a topological overlap matrix (TOM). Modules were visually delineated and labeled with distinctive colors, accompanied by the extraction of module features (MEs). Following network construction, the study assessed the relationship between modules and clinical features by calculating the Pearson correlation coefficient to gauge the strength of correlation between module expression patterns and clinical traits. Key module genes were identified by pinpointing modules exhibiting pronounced positive and negative correlations in the context of the module-trait relationship.

### Functional enrichment analysis

To determine the underlying biological processes and specific mechanisms by which pathogenic genes are associated with AP in patients with a BMI>30, GO and KEGG enrichment analyses of the CDEGs were performed. These CDEGs were the intersection of DEGs, key module genes, and LMRGs. The outcomes were depicted utilizing the "ggplot2" R library, and statistical significance was attained when the *p* value was less than 0.05 (*P* < 0.05). This comprehensive analytical framework provides valuable insights into the functional relevance and molecular pathways underlying AP in patients with a BMI>30, shedding light on the intricate mechanisms driving this condition.

### Immune infiltration analysis

To evaluate the degree of immune cell infiltration within the gene expression profiles linked to AP in patients with a BMI>30, this study employed the “GSVA” R library. Subsequently, the “ggplot2” R library, which is visually represented as a bar graph, was used to determine the abundance and proportion of infiltrating immune cells in each sample. To ascertain statistically significant differences in the proportions of 28 distinct immune cell types between the experimental group and the control group, Student's t test was conducted, considering a *p* value threshold of less than 0.05 (*P* < 0.05) to denote statistical significance.

### Machine learning algorithms

To detect potential biomarkers for AP among patients with a BMI>30, this study utilized the least absolute shrinkage and selection operator (LASSO), random forest (RF), and support vector machine recursive elimination (SVM-RE) algorithms to obtain genes with the greatest diagnostic value. Upon determining the intersecting genes, the study designated these overlapping entities as the hub genes, offering the most significant diagnostic value for AP in individuals with a BMI>30.

### Bayesian colocalization analysis

The assessment involved evaluating the likelihood that a single genetic variant contributes to variations in both the risk of AP and *HADH* expression, as well as affecting BMI and *HADH* expression, based on Genome-Wide Association Studies (GWAS) and expression Quantitative Trait Loci (eQTL) data [16, 17]. A posterior colocalization probability (PP4) of 80% was established as the threshold to indicate a shared causal signal. This shared causality was visualized using "LocusCompareR" [18], a tool designed for such comparative genomic analyses.

### External dataset validation

To enable cross-species analysis and further validate the findings, this study employed the "homologene" package within R software for the homologous transformation of hub genes into their corresponding mouse gene counterparts. Following this transformation, external datasets were utilized to validate the expression levels of these hub genes. This validation process is crucial for confirming the relevance and significance of the identified hub genes in different biological contexts, thereby enhancing the credibility and robustness of the study's findings.

### Establishment of a mouse model for high BMI-related AP

**Fig. 2:**
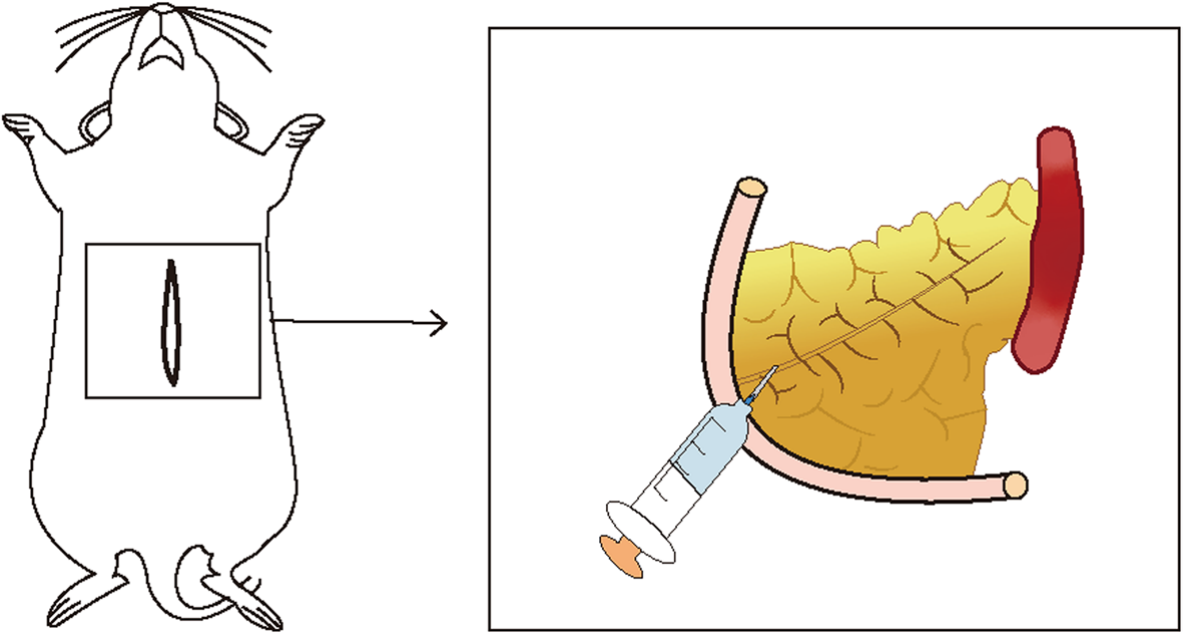
Modeling diagram. After the mice were anesthetized, the abdomen was disinfected with a cloth, the abdomen was opened layer by layer, the pancreas was exposed, the pancreatic duct was located, and sodium taurocholate or physiological saline was injected

In this study, eight-week-old male mice were maintained on a high-fat diet for 12 weeks. Following this period, six mice were randomly divided into two groups: a control group and an AP group. For the AP group, the mice were anesthetized and weighed, and their abdomens were sterilized. Surgical procedures were then performed to expose the pancreas and identify the pancreatic duct. A 5% sodium taurocholate solution was administered into the pancreatic duct at a dosage of 0.1 ml per 100 grams of body weight (Fig. 2). Conversely, the control group received a similar volume of physiological saline. All mice were euthanized 24 hours posttreatment, and blood samples were taken from the ophthalmic artery to measure pancreatic amylase and lipase levels. Pancreatic tissues were also collected for quantitative real-time polymerase chain reaction (q-PCR) analysis to further investigate the effects.

### qPCR

Total RNA was isolated from mouse pancreatic tissue specimens, and the RNA concentration was assessed utilizing an RNA purification kit. Next, RNA samples were reverse transcribed into cDNA with a reverse transcription kit. Subsequently, the polymerase chain reaction (PCR) protocol was applied, and the outcomes were evaluated utilizing the 2-ΔΔCt method. The primer sequences utilized in this investigation are listed in Table S1.

## Statistical analysis

All the statistical analyses and visualizations were performed using R software (version 4.3.1). Comprehensive descriptions of the statistical tests employed can be found in the corresponding bioinformatics methods section and figure legends.

## Results

### Causal effect of BMI on AP

**Fig. 3:**
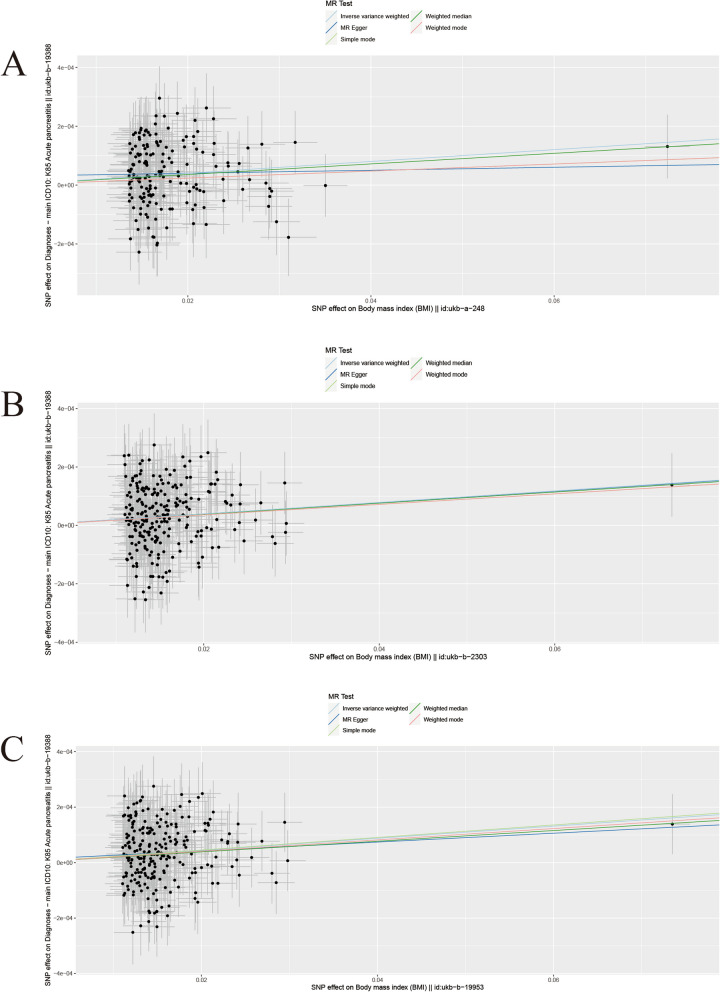
Scatter plots of causality in AP on 3 BMI datasets. The slope of each line corresponding to the estimated MR effect in different models. **A** ukb-a-248. **B** ukb-b-2303. **C** ukb-b-19953

Across all three datasets, there was no significant evidence of horizontal pleiotropy or heterogeneity, leading us to select the inverse variance weighted (IVW) method as our primary analytical technique. The findings, detailed in Table S2 and illustrated in Fig. 3, demonstrated a significant causal relationship between BMI and the likelihood of developing AP. Specifically, the genetic variants ukb-a-248 (*P* < 0.05, odds ratio [OR] 95% confidence interval [CI] = 1.0020 [1.0012–1.0029]), ukb-b-2303 (*P* < 0.05, OR 95% CI = 1.0020 [1.0011–1.0028]), and ukb-b-19953 (*P* < 0.05, OR 95% CI = 1.0022 [1.0013–1.0030]) were found to have a significant causal relationship with ukb-b-19388, indicating a positive association between BMI and the risk of AP. This observation was corroborated by a weighted median analysis, which served as a secondary analytical method and further supported the IVW results.

### Sensitivity analysis

**Fig. 4:**
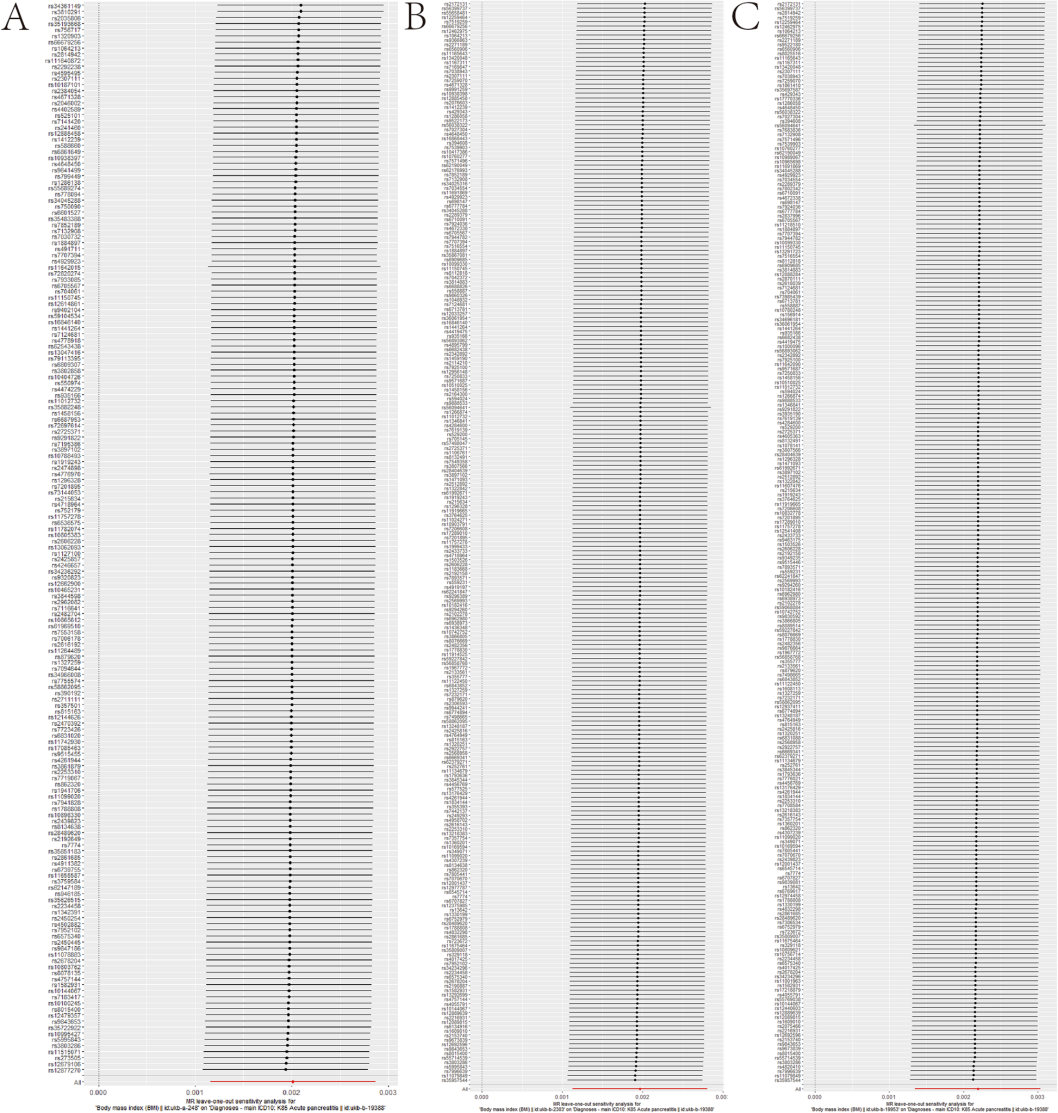
Leave-one-out sensitivity tests. The MR results of the remaining IVs were calculated after removing the IVs one by one. **A** ukb-a-248. **B** ukb-b-2303. **C** ukb-b-19953

In the sensitivity analysis, which involved excluding one SNP at a time, the study revealed that the relationship between specific BMI ranges and the likelihood of developing AP remained stable. This robustness check confirmed that the observed causal connection did not rely on any single genetic variant, thereby strengthening the reliability of the results (Fig. 4).

### Identification of obesity-related DEGs (ORDEGs)

**Fig. 5:**
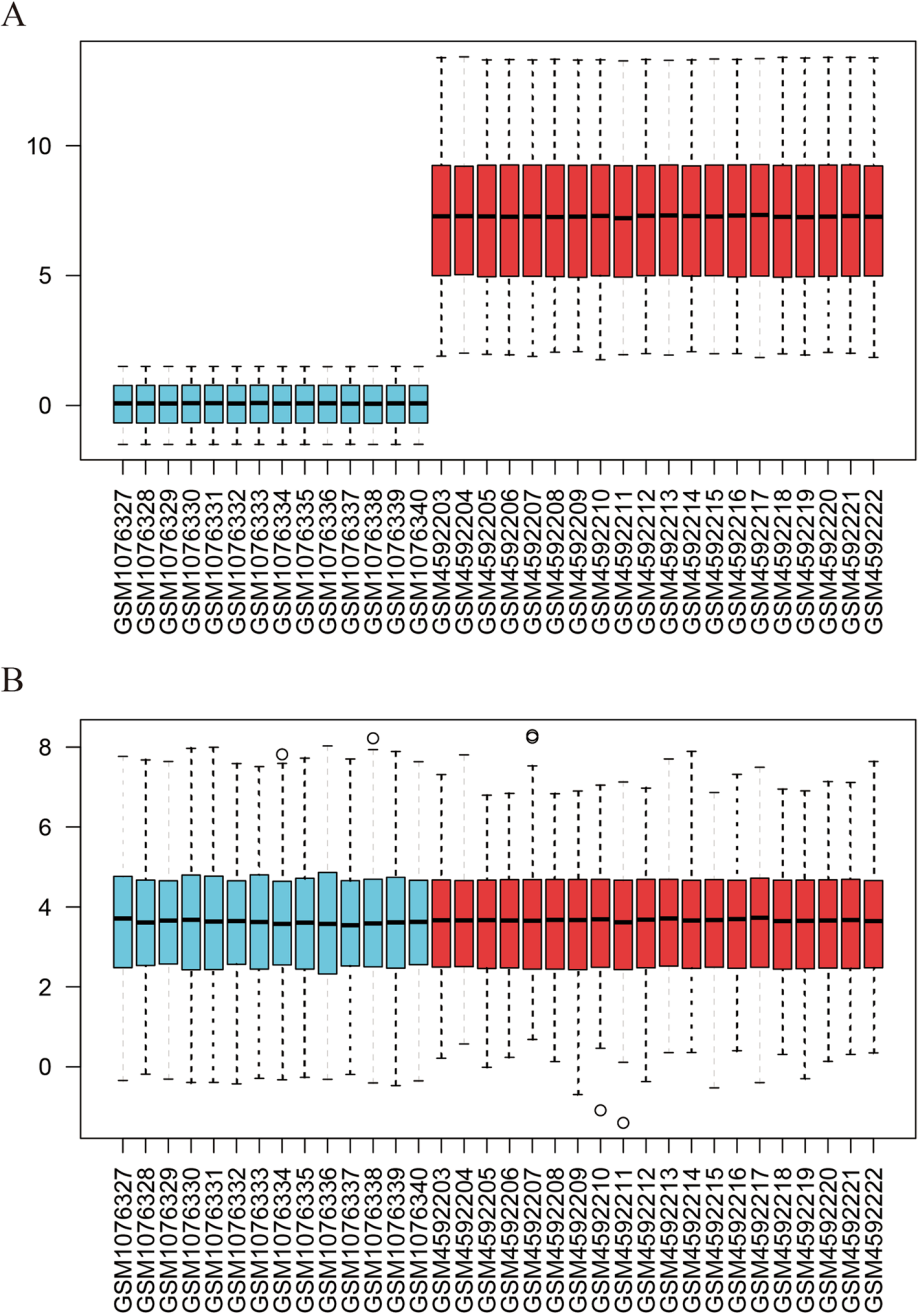
Boxplots of gene expression before and after standardization for 2 selected GEO datasets. **A** Before standardization. **B** After standardization

**Fig. 6:**
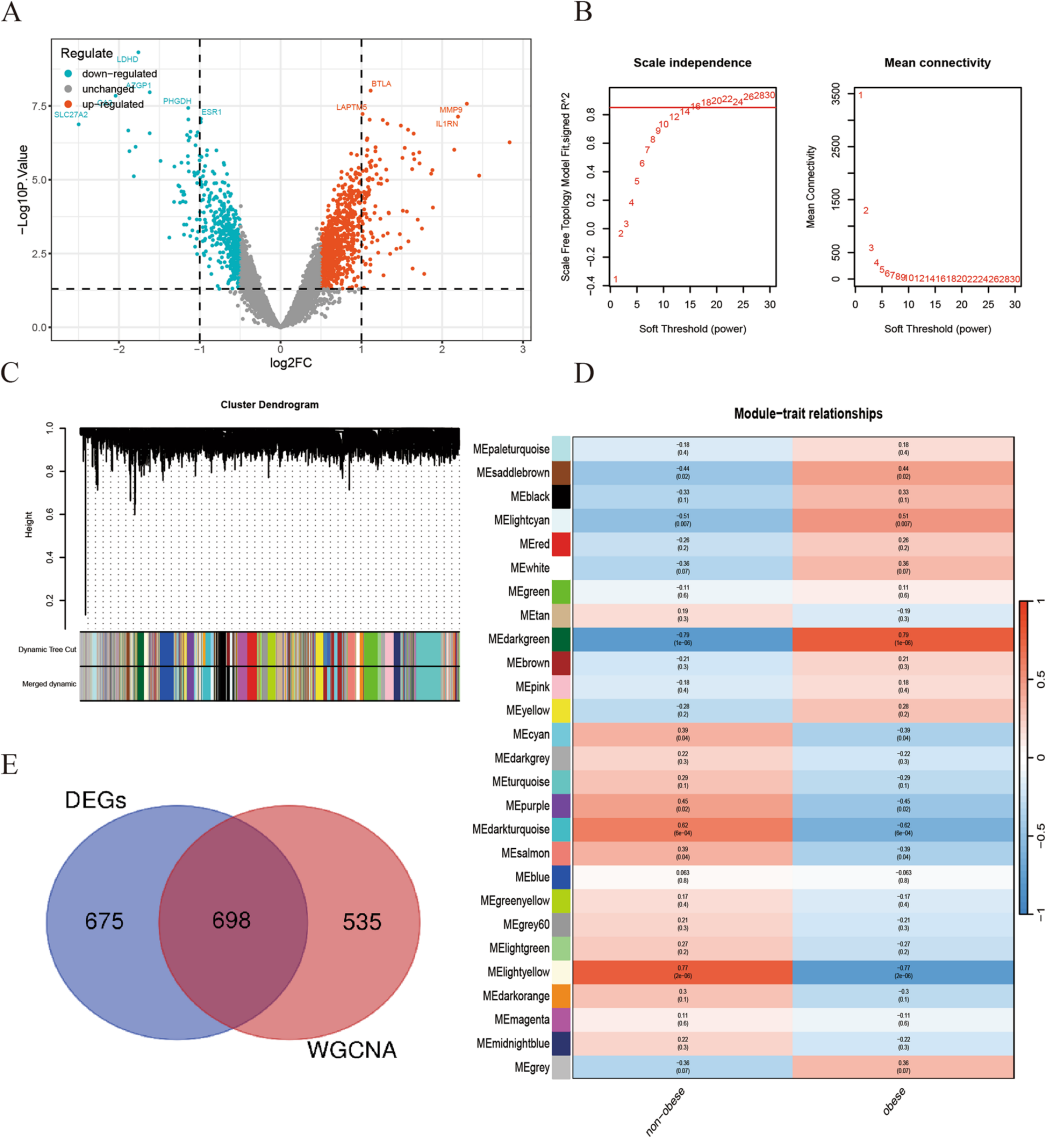
Identification of ORDEGs. **A** Volcano plot showing DEGs in the BMI>30 and BMI <30 samples. **B** Soft-thresholding filtering. **C** Clustering dendrogram of genes. **D** Correlation heatmap of gene modules and clinical features. **E** Venn diagram showing the overlap of module genes and DEGs

The expression levels before and after removing batch effects are depicted in Fig. 5. Postremoval, the data distribution across different datasets became notably more uniform, with medians aligned and both the mean and variance showing greater consistency. Subsequent to this adjustment, a differential expression analysis was conducted, identifying a total of 1,372 DEGs in the experimental samples, which included 531 genes that were downregulated and 841 genes that were upregulated. The top 10 genes (*RFX7, CSTA, TXN, TRIP4, IFNGR2, ATP6V1E1, GTF2B, CSF2RA, GMFG* and *GLT1D1*) with the most significant differences in expression are highlighted in Fig. 6A. Subsequently, WGCNA was utilized to detect coexpressed gene modules within both the BMI>30 and BMI<30 groups. To ensure adherence to scale-free network criteria, a soft threshold of β=16 was selected, as indicated by a scale-free R2 value of 0.85 (Fig. 6B). The dynamic tree cut algorithm successfully delineated 27 distinct gene modules (Fig. 6C). Of particular interest, the light yellow, dark turquoise, and dark green modules exhibited a pronounced correlation with BMI>30, demonstrating a strong correlation coefficient (|R| > 0.6) and significance level (*P* < 0.01) (Fig. 6D). These modules collectively encompassed 1233 genes. Finally, from these modules, a subset of 698 ORDEGs were selected for further investigation (Fig. 6E)

### Identification of AP-related DEGs (APRDEGs)

**Fig. 7:**
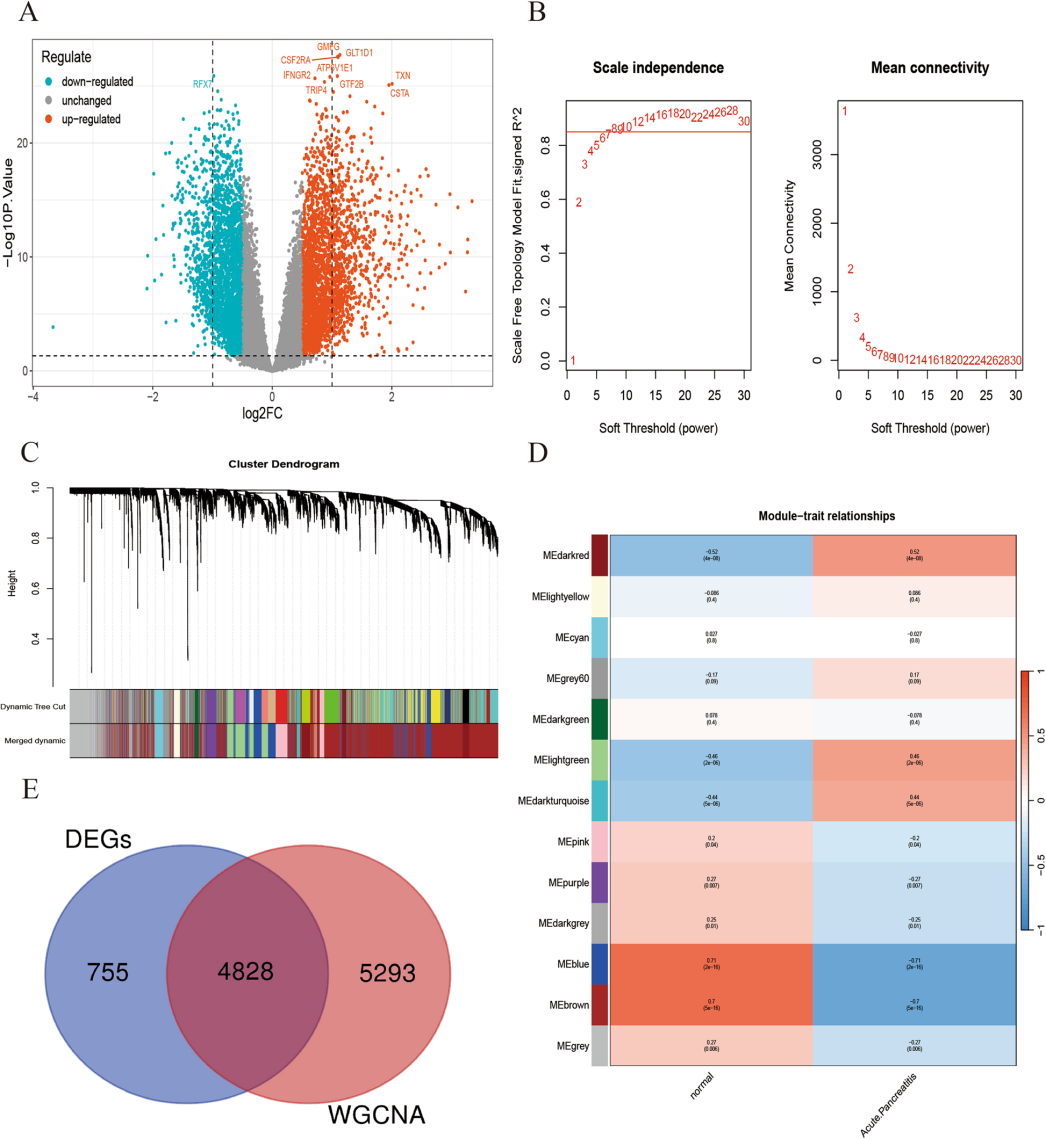
Identification of DEGs and AP-related module genes. **A** Volcano plot showing DEGs in the AP and normal samples. **B** Soft-thresholding filtering. **C** Clustering dendrogram of genes. **D** Correlation heatmap of gene modules and clinical features. **E** Venn diagram showing the overlap of module genes and DEGs

In the AP dataset, a total of 5,582 genes were differentially expressed among the experimental samples, with 2,885 genes downregulated and 2,697 genes upregulated. The top 10 genes (*LDHD, AZGP1, CA3, SLC27A2, PHGDH, ESR1, BTLA, MMP9, LAPTM5* and *IL1RN*) exhibiting the most significant differences in expression are highlighted in Fig. 7A. Subsequently, WGCNA was employed to detect coexpressed gene modules within both the experimental and control cohorts. To maintain compliance with scale-free network standards, a soft threshold of β= 8 was chosen, as indicated by a scale-free R2 value of 0.85 (Fig. 7B). The dynamic tree cut algorithm successfully delineated 27 distinct gene modules (Fig. 7C). Interestingly, the blue and brown modules exhibited a pronounced correlation with obesity, demonstrating a strong correlation coefficient (|R| > 0.6) and significance level (*P* < 0.01) (Fig. 7D). These modules collectively encompassed 10121 genes. Finally, from these modules, a subset of 4828 DEGs was selected for further investigation (Fig. 7E). LMRGs, ORDEGs and APRDEGs were defined as common differentially expressed genes (CDEGs).

### Enrichment analysis of CDEGs

**Fig. 8:**
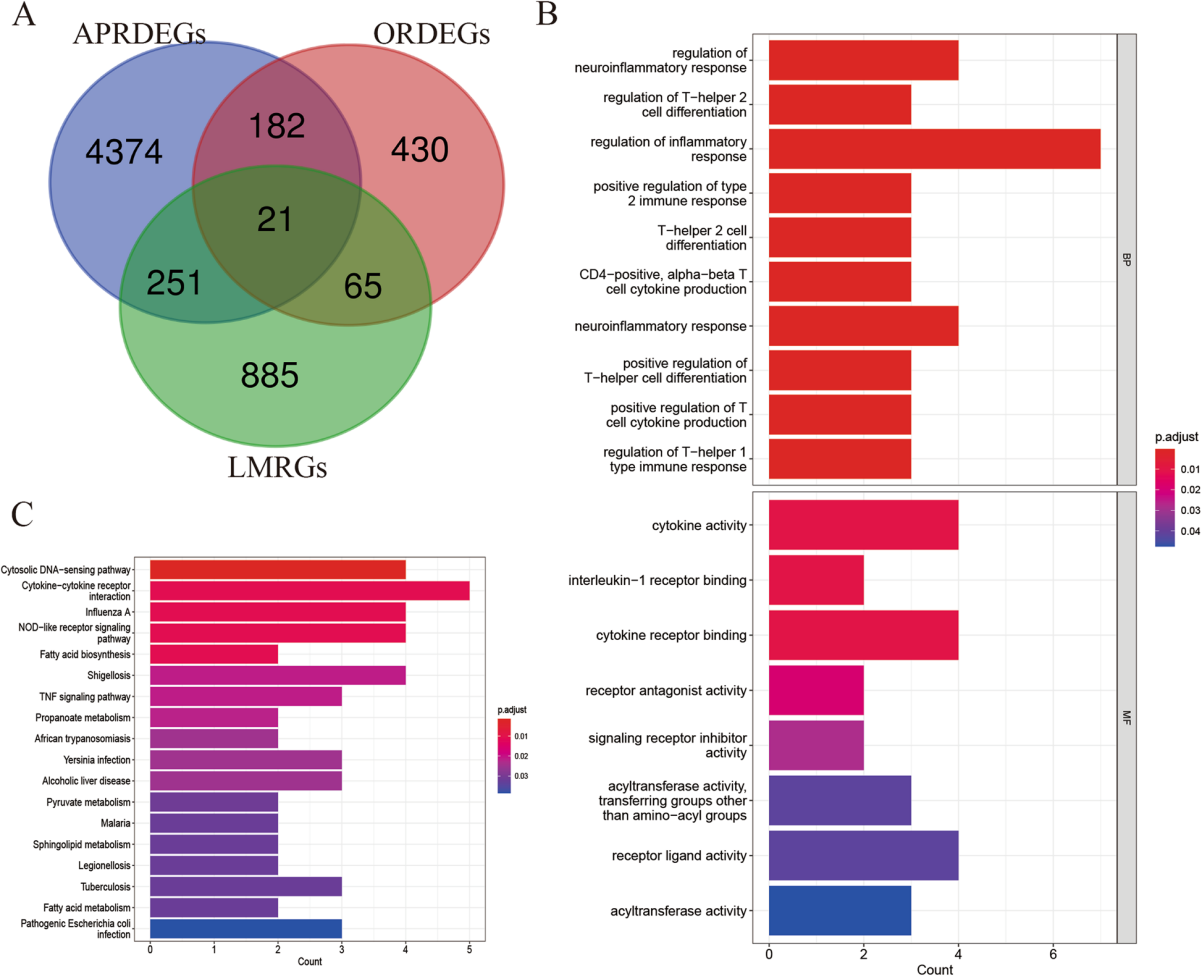
Enrichment analysis of the intersecting genes. **A** A total of 21 overlapping genes were identified among the APRDEGs, ORDEGs and LMRGs. **B** Gene Ontology (GO) enrichment results of 21 intersecting genes. **C** Kyoto Encyclopedia of Genes and Genomes (KEGG) enrichment results for 21 intersecting genes

Twenty-one CDEGs were screened, which were potential marker genes for AP in patients with a BMI>30 based on lipid metabolism (Fig. 8A). GO and KEGG enrichment analyses were performed on the 21 CDEGs (*ABCA3, NLRP3,FASN, ORMDL3, SERPINA1, SPHK1, IL18, ESR1, LRIG1, CHKA, ACSS2, HADH, SGMS2, IL1RN, IL4R, CCL5, ACACB, IL1B, HSDL2, MBOAT2* and *MMP9*) mentioned above to explore common regulatory pathways. GO analysis suggested that shared genes may be related to the regulation of the neuroinflammatory response, regulation of T-helper 2 cell differentiation and regulation of the inflammatory response (Fig. 8B). The KEGG analysis suggested that these genes might be primarily associated with the cytosolic DNA−sensing pathway and cytokine−cytokine receptor interaction (Fig. 8C).

### Immune cell landscape

**Fig. 9:**
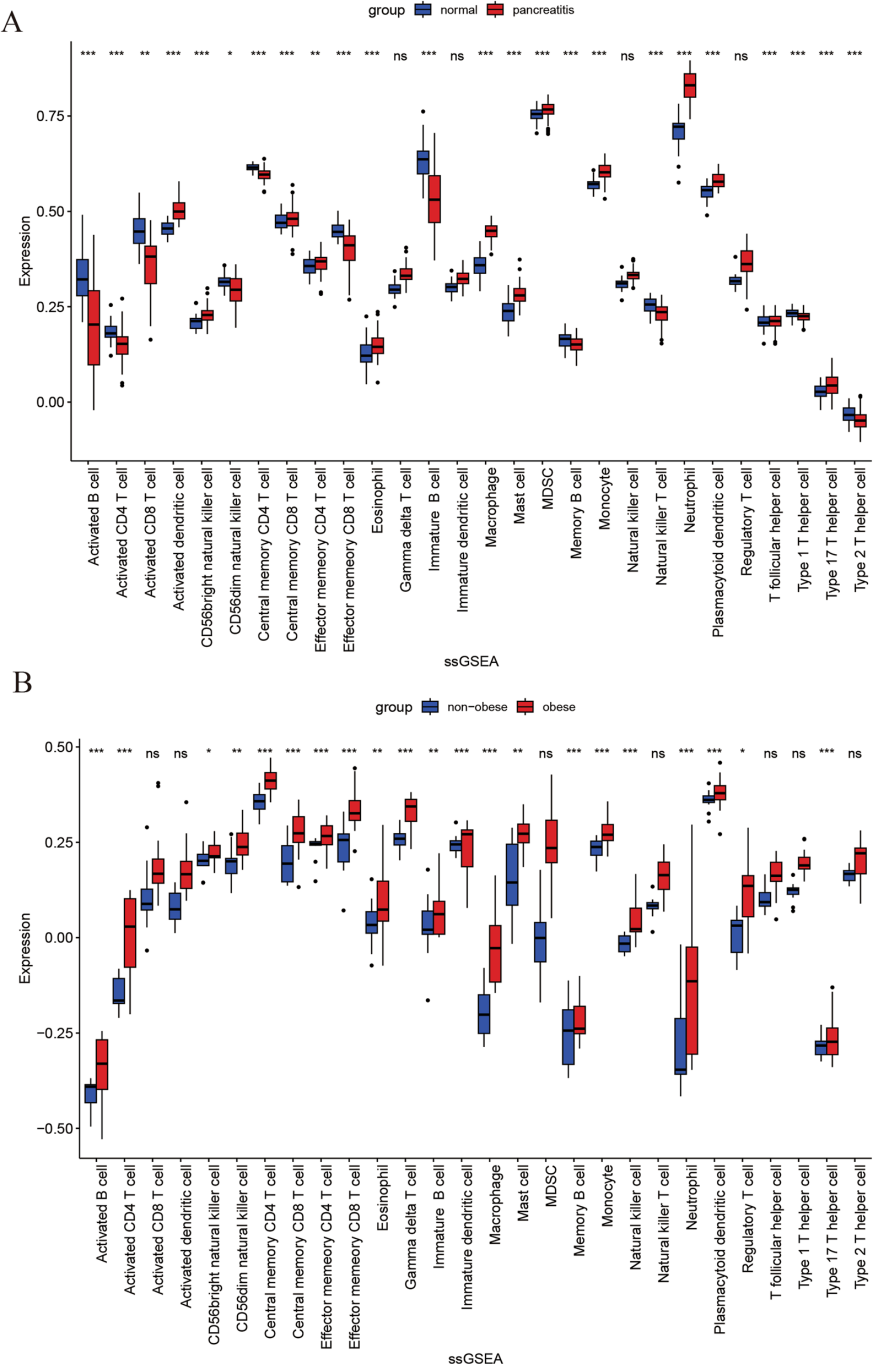
Box diagram of the proportions of 28 types of immune cells. **A** dataset with a BMI >30 showed a difference in infiltration between the two groups. **B** AP dataset showing the difference in infiltration between the two groups

To investigate the pivotal function of immune cells in the onset and progression of AP among patients with a BMI>30, this study separately assessed immune infiltration levels in both the BMI>30 and AP datasets. In the AP dataset, 24 out of 28 types of immune cells exhibited noteworthy variances, all displaying elevated expression levels in the AP group (Fig. 9A). Conversely, within the BMI>30 dataset, 24 out of the 28 cell types analyzed showed significant differences, as depicted in Fig. 9B. Among these, activated dendritic cells, CD56bright natural killer cells, central memory CD8+ T cells, effector memory CD4+ T cells, eosinophils, and macrophages demonstrated consistent trends.

### Identification of key genes by machine learning algorithms

To systematically filter out nonessential genes, this study employed three distinct machine learning techniques to identify pivotal genes within the BMI>30 and AP datasets separately.

**Fig. 10:**
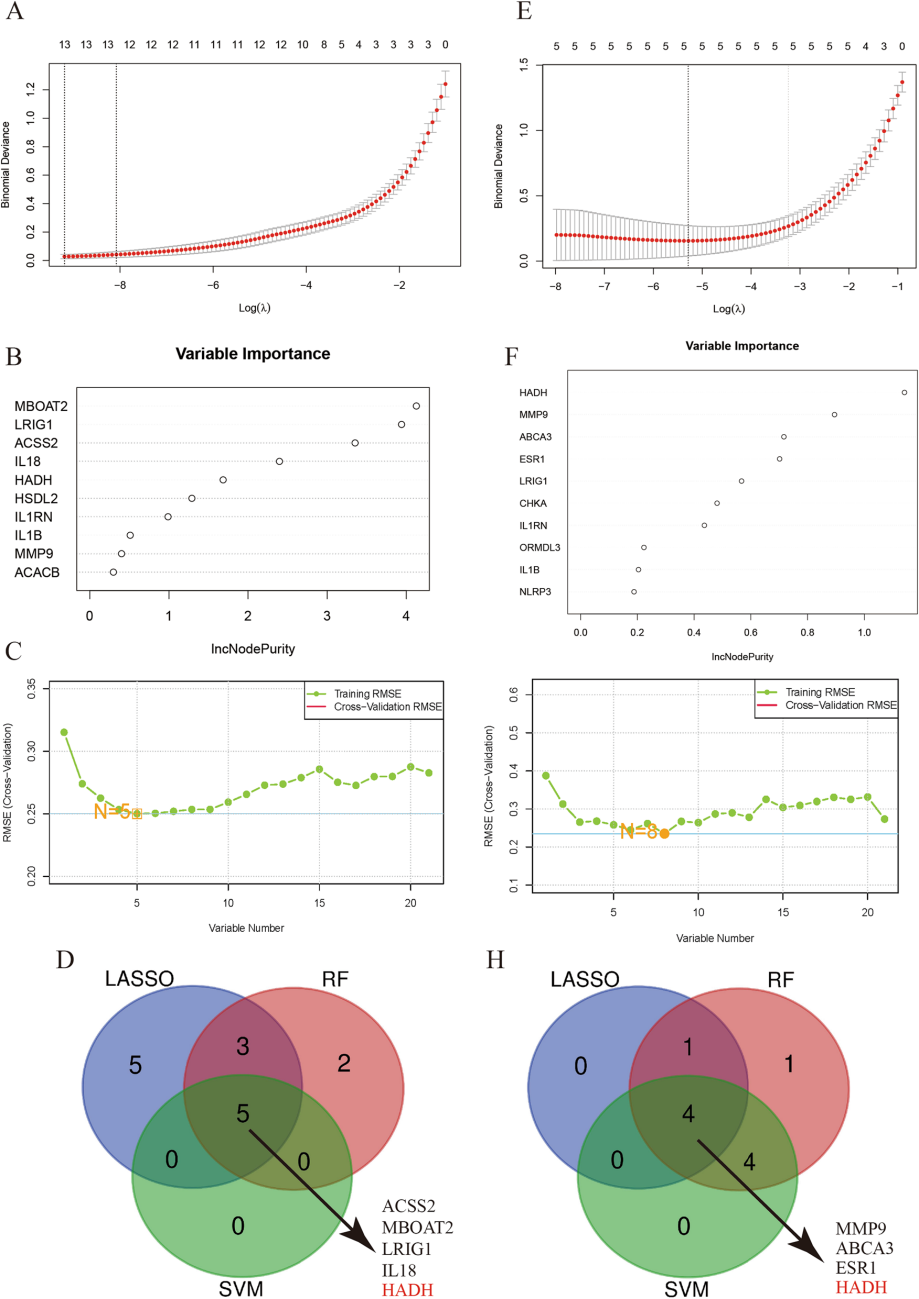
Selection of potential diagnostic biomarkers with machine learning methods. **A** LASSO regression analysis was applied to screen diagnostic biomarkers based on the 21 intersecting genes in the AP dataset. The genes with the lowest binominal deviance were identified as the most suitable candidates. **B** The results of the Gini coefficient method for the random forest classifiers in the AP dataset. The x-axis represents genetic variables, and the y-axis represents importance indices. **C** The number of CDEGs with the lowest error and highest accuracy were considered the most suitable candidates via the SVM-RFE algorithm in the AP dataset. **D** Venn diagram visualizing the overlap of selected biomarkers between 3 algorithms, yielding 5 genes selected as candidate biomarkers. **E** LASSO regression analysis was applied to screen diagnostic biomarkers based on the 21 intersecting genes in the BMI>30 dataset. The genes with the lowest binominal deviance were identified as the most suitable candidates. **F** The results of the Gini coefficient method for the random forest classifiers in the BMI>30 dataset. The x-axis represents genetic variables, and the y-axis represents importance indices in the BMI>30 dataset. **G** The number of CDEGs with the lowest error and highest accuracy were considered the most suitable candidates via the SVM-RFE algorithm in the AP dataset. **H** Venn diagram visualizing the overlap of selected biomarkers between 3 algorithms, yielding 4 genes selected as candidate biomarkers

For the AP dataset, LASSO regression was initially utilized to screen 13 genes (*FASN, SERPINA1,SPHK1,IL18, LRIG1, CHKA, ACSS2, HADH, SGMS2, IL4R, ACACB, HSDL2* and *MBOAT2*) from a pool of 21 CDEGs (Fig. 10A). Subsequently, the RF algorithm was applied, revealing 10 genes (*MBOAT2, LRIG1, ACSS2, IL18, HADH, HSDL2,* *IL1RN,* *MMP9,* *ACACB* and *SERPINA1*) of significance (Fig. 10B), while the SVM-RFE algorithm identified 5 genes (*ACSS2, MBOAT2, LRIG1, IL18* and *HADH*) (Fig. 10C). These outcomes were then combined, resulting in the identification of the final 5 genes out of 21 (*ACSS2, MBOAT2, LRIG1, IL18, HADH*) as potential biomarkers for AP (Fig. 10D).

In the BMI >30 dataset, LASSO regression pinpointed 5 genes (*ABCA3,* *NLRP3, ESR1, HADH* and *MMP9*) from the initial pool of 21 CDEGs (Fig. 10E). Additionally, the RF algorithm identified 10 genes (*HADH, MMP9, ABCA3, ESR1, CHKA, LRIG1, IL1RN, ORMDL3, IL1B* and *NLRP3*) (Fig. 10F), and the SVM-RFE algorithm highlighted 8 genes (*MMP9, HADH, ESR1, ABCA3, IL1RN, LRIG1, CHKA and IL1B*) (Fig. 10G). Following the convergence of these results, 4 of the 21 genes (*MMP9, ABCA3, HADH, and ESR1*) were identified as potential biomarkers for BMI>30 (Fig. 10H). Ultimately, this study tentatively identified *HADH* as the most crucial biomarker for AP in patients with a BMI>30.

### Colocalization results

In this investigation, a comprehensive analysis was conducted to ascertain the likelihood of a shared genetic variant among four GWAS datasets—three related to BMI and one related to AP—in conjunction with the eQTL of the *HADH* gene, a scenario designated as PP4. Our findings substantiate the pivotal influence of the *HADH* gene on variations in BMI and susceptibility to AP, as evidenced by PP4 values of 100.00%, 98.58%, 98.61%, and 98.61% for each dataset, respectively (Fig. S1).

### Validation of *HADH*

To assess the potential utility of *HADH* in diagnosing AP among patients with a BMI >30, this study conducted validation experiments on mice and analysed the results using training datasets (GSE109227 and GSE166047).

**Fig. 11:**
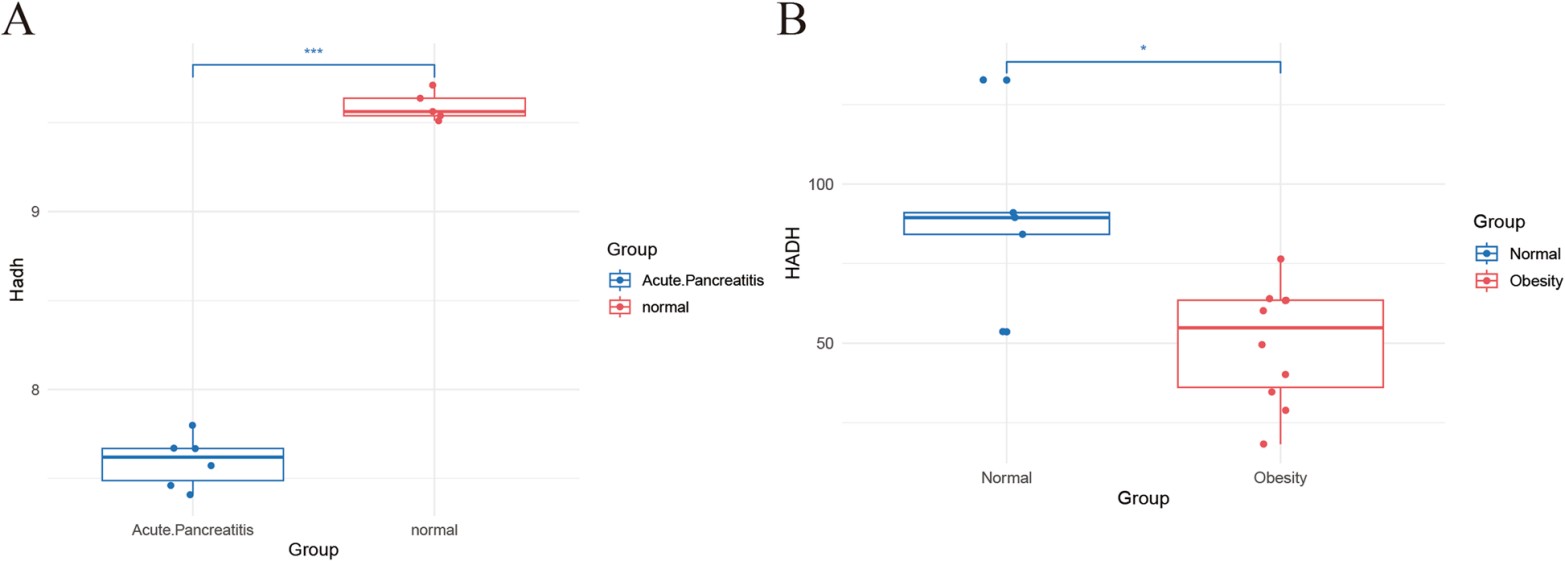
Diagnostic values of the candidate biomarkers BMI>30 and AP assessed by expression comparison. **A** Comparison of *HADH* gene expression between the AP and normal groups in the GSE109227 test dataset. **B** Comparison of *HADH* expression between the BMI >30 and BMI< 30 groups in the GSE166047 test dataset (* *P* < 0.05, *** *P* < 0.001)

In the GSE109227 dataset, a statistically meaningful variance (*P* < 0.0001) in the expression of *Hadh*, an *HADH* homolog in mice, was observed (Fig. 11A). Similarly, in GSE166047, *HADH* exhibited a statistically meaningful variance (*P* < 0.05) in expression levels between samples with a BMI >30 and those with a BMI< 30 (Fig. 11B).

### Validation of *HADH* Using qPCR

**Fig. 12:**
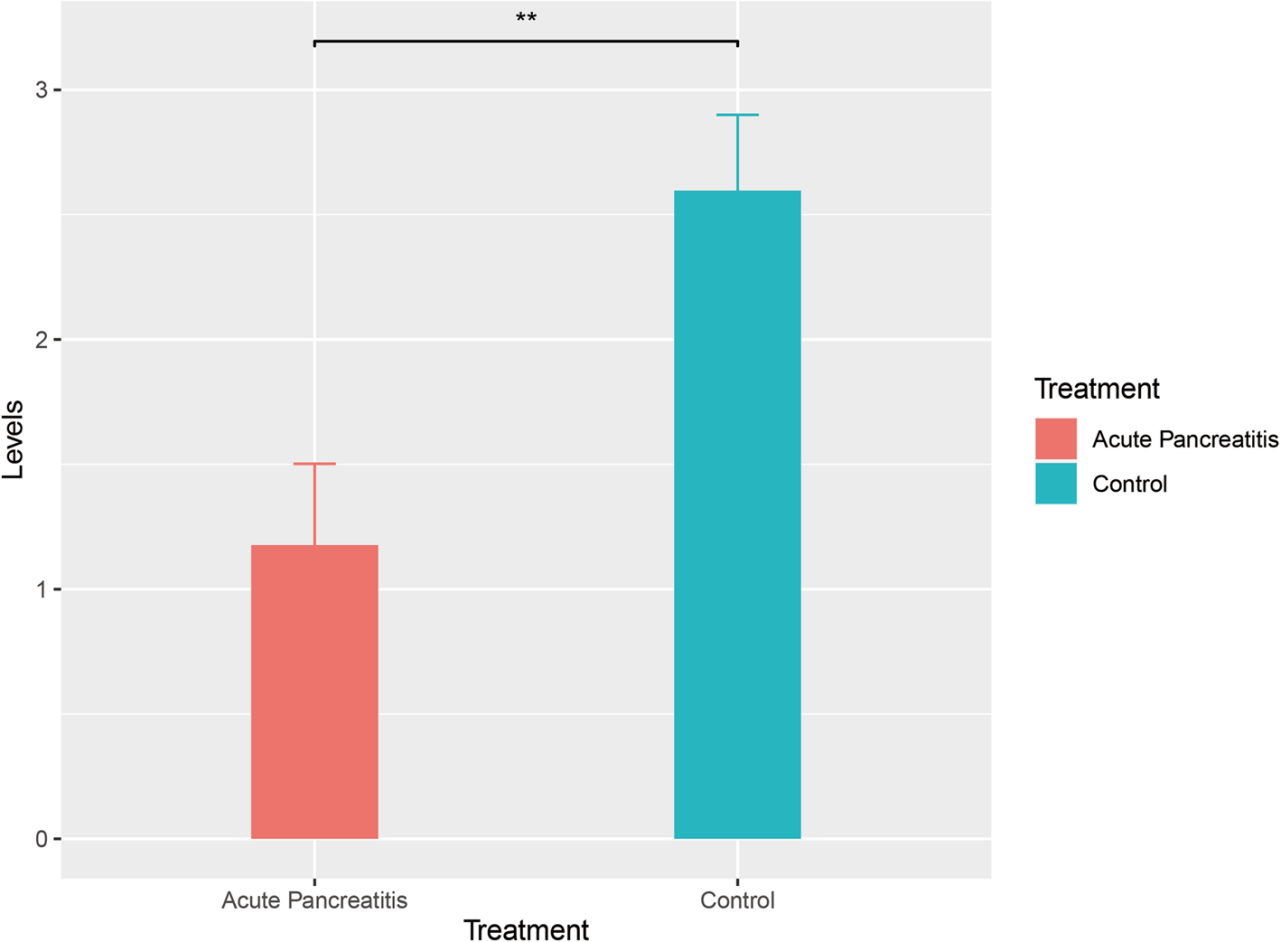
The results of q-PCR analysis of mRNA expression levels are shown. The expression levels of *HADH* in patients with a high BMI were significantly greater than those in patients with AP and a high BMI (** *P* < 0.01)

The qPCR results revealed a notable decrease in *HADH* expression in the AP cohort with a high BMI compared to that in the control group (*P* < 0.05) (Fig. 12). Clinical attributes, including pancreatic amylase, pancreatic lipase, and other pertinent parameters for the six mice, are detailed in Table S3. In summary, *HADH* has emerged as a prospective biomarker for diagnosing AP in patients with a BMI>30.

## Discussion

The global prevalence of AP substantiates its significance as a common gastrointestinal emergency, necessitating urgent attention and intervention. Although substantial advancements have been made in the clinical treatment and diagnostic laboratory parameters of AP in recent years, the intricate anatomical positioning of the pancreas, coupled with the subtle presentation of this type of pancreatitis, poses significant challenges in the realm of early diagnosis. Delayed detection of the disease has profound ramifications, including heightened susceptibility to complications and elevated mortality rates. Failing to promptly diagnose and institute appropriate therapeutic measures has led to severe pancreatitis in a substantial portion of patients, such as necrotizing pancreatitis or even organ failure. Consequently, the elucidation of diagnostic biomarkers for this condition remains a pressing concern. Mounting evidence points to a conspicuous correlation between high BMI and the onset and progression of AP, as highlighted in the literature [19, 20]. An increased prevalence of comorbidities is frequently observed in obese patients, predisposing them to an elevated risk of developing severe pancreatitis [5, 21]. Furthermore, lipids, which are essential elements of cellular architectures, play a pivotal role in forming phospholipid bilayers, which are fundamental to cell membrane integrity and function. In addition to serving as vital sources of energy and key players in cellular metabolic activities, rigorous scientific investigations have revealed the complex dynamics interlinking lipid metabolism with obesity and AP. These studies have shed light on the profound and intricate connections underpinning these biological phenomena, offering new insights into their interdependencies [7, 12, 22, 23]. In the pursuit of tailored diagnostic and therapeutic approaches for AP, it has become crucial to investigate the cumulative effect of BMI > 30, AP, and lipid metabolism from the standpoint of gene expression.

This study broke new ground by utilizing available GWAS data through a dual-sample MR approach to clarify the causal link between BMI and the risk of AP. The results decisively demonstrated a direct correlation between higher BMI and increased vulnerability to AP. In an effort to explore the molecular foundations of AP, especially among individuals with a BMI > 30, within the context of lipid metabolism, this research undertook a series of critical analytical efforts. The “limma” R package offers a robust framework for analysing gene expression data [15]. In this study, 1372 DEGs were observed among the BMI > 30 and BMI < 30 groups using the “limma” R package. In this study, 1233 significant module genes were identified, 698 ORDEGs were identified from the intersection of DEGs and module genes. Subsequently, the same methods were used to analyse the AP dataset, resulting in 4828 APDEGs. The intersection of ORDEGs and APDEGs, along with LMRGs, revealed 21 common risk genes associated with AP in patients with a BMI > 30. This foundational phase of this study enabled the identification of genes that undergo significant expression changes, potentially playing pivotal roles in the pathogenesis of AP among individuals with a BMI > 30. Despite the recognition of these genes, the precise mechanisms through which they contribute to AP regulation in obese patients remain elusive. Moreover, the biomarkers initially identified, while numerous, proved to be impractical for clinical application. The objective of this investigation was to refine the search for diagnostic biomarkers with increased specificity and accuracy, necessitating a more detailed examination of the expression of these genes.

Initially, to elucidate the regulatory mechanisms of the identified genes within the human body, this study performed an enrichment analysis of signaling pathways and biological functions linked to the 21 identified genes. KEGG analysis revealed that these CDEGs were predominantly enriched in pathways and functions associated with infection and inflammation. These pathways included the cytosolic DNA-sensing pathway, cytokine‒cytokine receptor interaction pathway, and NOD-like receptor signaling pathway. These findings indicate that these terms are closely related to inflammatory processes and the body's response to them. Prior research has underscored the pivotal role of cytosolic DNA sensing in tissue damage and inflammation across a variety of diseases [24–27]. Obesity-induced mitochondrial DNA (mtDNA) release initiates an increase in chronic sterile inflammatory responses in adipose tissue via this pathway [28]. Additionally, it plays a role in inflammation associated with AP, where acinar cell death activates interferon (IFN) signaling through the STING pathway in macrophages. This highlights the essential role of IFNs in AP through various innate immune-sensing pathways [29]. Cytokine‒cytokine receptor interactions and the MAPK signaling pathway are associated with consistent cytokine expression throughout different stages of severe acute pancreatitis, indicating their involvement in the regulation and progression of the inflammatory response during the disease [30]. NOD-like receptors constitute a broad and intricate group of signaling regulators. These proteins consolidate both favorable and unfavorable signals and subsequently activate additional signaling regulators implicated in inflammatory responses, tumorigenesis, cellular senescence, and stem cell characteristics [31]. Moreover, inhibition of the NLRP3 inflammasome has been shown to reduce the degree of experimentally induced AP in obese mice [32]. These findings shed light on the roles of these genes in contributing to the intricate regulatory networks that oversee cellular functions and systemic responses.

Moreover, GO analysis revealed significant enrichment in processes such as the neuroinflammatory response, regulation of T-helper 2 cell differentiation, regulation of the inflammatory response, and positive regulation of the type 2 immune response. We were surprised to find that the GO terms were predominantly related to immunity and inflammation. To gauge the extent of infiltration by immune elements and understand the proportion of immune cells in AP in patients with a BMI > 30, this study employed the "ssGSEA" algorithm to assess immune infiltration levels. Several immune cell types, such as activated dendritic cells, CD56bright natural killer cells, central memory CD8+ T cells, effector memory CD4+ T cells, eosinophils, and macrophages, exhibited consistent correlations in the BMI>30 and AP datasets. These findings significantly underscore the role of immune factors in the mechanisms triggering AP in individuals with a BMI > 30. These findings suggest the initiation of a prolonged proinflammatory response and the mobilization of inflammatory cells, consistent with the findings of previous studies [33–35]. These findings offer valuable insights, indicating that inflammation plays a crucial role in the pathogenesis of AP in patients with a BMI > 30.

To pinpoint more accurate diagnostic biomarkers for AP in patients with a BMI > 30, focusing on lipid metabolism, this study employed LASSO, RF, and SVM-RFE to conduct further analysis on two disease-specific datasets. LASSO regression, commonly used to filter variables and mitigate the risk of overfitting, was employed. The optimal number of DEGs can be determined using the binomial deviation method [36]. RF is adept at ranking genes and is well suited for managing high-dimensional data, constructing prognostic models, and assessing the significance of individual variables [37]. SVM-RFE has proven to be a convenient tool for eliminating redundant components and retaining outcome-relevant variables, especially in datasets with limited samples [38]. In this study, four genes (*MMP9, ABCA3, HADH* and *ESR1*) were identified as potential diagnostic markers by cross-referencing the outcomes derived from three distinct machine learning methodologies in the BMI >30 dataset. The same methodologies were applied to pinpoint five genes (*ACSS2, MBOAT2, LRIG1, IL18* and *HADH*) with potential diagnostic value in the AP dataset. Fascinatingly, the *HADA* gene has prominently surfaced during the meticulous search for diagnostic biomarkers applicable to both conditions under study. This finding was validated through subsequent colocalization analysis, analysis of GEO external datasets, and qPCR experiments in animal models. This observation led us to propose that the HADA gene has a significant, undeniable influence on the initiation and progression of AP in individuals with a BMI >30.

In the context of recent advancements, an extensive body of work has been devoted to pinpointing biomarkers critical for the diagnosis and progression of AP. A landmark study in 2023 by Zheng Wang et al. revealed severe AP, with a special focus on the role of immunogenic cell death mechanisms. Their groundbreaking findings revealed that *LY96, BCL2,* and *IFNGR1* were instrumental biomarkers for both the emergence and evolution of severe AP [35]. In a hospital-based case‒control investigation, Francisco D'Oliveira Martins and his team proposed that *GSTM1* may increase vulnerability to AP [39]. Data from the AP, septic AP, and control groups were collected, and CitH3 levels were meticulously quantified using enzyme-linked immunosorbent assay (ELISA). This method is pivotal for identifying circulating CitH3 as a reliable marker for diagnosing and predicting outcomes in septic AP patients [40]. A subsequent forward-looking, double-blind study identified fatty acid ethyl ester (FAEE) as a precise marker for diagnosing alcohol-related pancreatitis [41]. Further research has highlighted the potential of intercellular adhesion molecule 1, red cell distribution width (RDW), along with urinary trypsinogen-2 and trypsinogen activating peptide (TAP), as informative biomarkers for AP [42–44]. Despite these advances, investigations specifically targeting AP in individuals with a BMI >30 are lacking. Bridging this knowledge gap, our research suggested that *HADH* is a novel biomarker indicative of AP onset within this specific population.

*HADH* is located on chromosome 4q25 and is affiliated with the 3-hydroxyacyl-CoA dehydrogenase gene family. It codes 3-hydroxyacyl-CoA dehydrogenase, a pivotal enzyme in the fatty acid beta-oxidation pathway. *HADH* expression is widespread across various tissues (especially adipose tissue), with notably high enzyme activity observed in the pancreas. Mutations in *HADH* have been linked to hyperinsulinemic hypoglycemia, a condition characterized by abnormalities in insulin secretion and recognized as a fatty acid oxidation deficiency disease [45–48]. Moreover, reduced *HADA* expression has been demonstrated to enhance tumor cell migration and invasion by activating the Akt signaling pathway [49]. Research indicates that elevated *HADH* expression is correlated with an unfavorable prognosis in acute myeloid leukemia patients [50]. Poor clinical outcomes have also been observed in colon cancer patients with high *HADH* expression [51]. These findings highlight the diverse roles of *HADA* in cellular processes across different diseases.

A study leveraging proteomics techniques revealed that, in comparison with individuals of normal weight, obese patients exhibited markedly lower *HADH* protein levels, averaging merely 45% of the control group's *HADH* protein levels. Analysis via Ingenuity Pathway Analysis suggested that this decrease in *HADH* might be associated with inhibited activation of the LXR/RXR pathway [52]. This hypothesis is supported by experimental observations in which *HADH*-deficient mice displayed a compromised ability to metabolize TG in plasma under cold stress conditions. This impairment led to significant triglyceride and fatty acid accumulation [53], underscoring the role of elevated plasma fatty acid levels in promoting obesity. Fatty acid interferes with the ability of insulin to inhibit lipolysis [54], leading to increased fatty acid circulation and accumulation, which in turn triggers the proinflammatory NF-κB pathway in both animal models and cell culture studies, indicating persistent inflammation [55]. Moreover, *HADH* is crucial for the differential handling of stored lipids [56], highlighting its significant role in metabolic health and disease progression.

What series of reactions occur in AP patients with a BMI >30 due to alterations in *HADH* expression levels? In patients exhibiting a high BMI, the downregulation of *HADH* expression reduces fatty acid beta-oxidation, the primary pathway for fatty acid degradation, causing intracellular fatty acid accumulation. This metabolic alteration has profound implications for cellular physiology: (1) Fatty acid accumulation can lead to mitochondrial dysfunction, selectively inhibiting the active form of mitochondrial complex I. This suppression triggers necrotic cell death by releasing intracellular calcium and disrupting mitochondrial complexes I and V [57, 58]. These mitochondrial dysfunctions have extensive implications, disrupting cellular energy homeostasis and overall functionality. (2) The increase in free fatty acids subsequently triggers an increase in reactive oxygen species (ROS) production. ROS act as potent mediators of mitochondrial damage and tissue inflammation, contributing to the pathogenesis of AP [59]. (3) Elevated levels of fatty acids decrease the production of reduced glutathione, further compromising the cell's capacity to mitigate oxidative stress [60]. (4) The buildup of free fatty acids within cells can induce lipotoxicity, resulting in local and systemic consequences. This plays a role in the inflammatory response, multisystem organ failure, and necrotic acinar cell death in AP among patients with a BMI >30 [58]. Inflammation is a significant consequence of disrupted fatty acid metabolism and mitochondrial dysfunction. The upregulation of inflammatory mediators stimulated by elevated fatty acid levels implies a potential connection between *HADH*-related pathways and the onset of inflammatory conditions. Additionally, other studies have confirmed that the release of free fatty acids contributes to the exacerbation and severity of AP [61]. Based on previous research findings and our own findings, this study revealed that reduced *HADH* expression disrupts lipid metabolism, leading to the accumulation of FFAs, potentially playing an essential role in the pathogenesis of AP individuals with a BMI >30. This underscores the importance of conducting further investigations in future studies.

To validate this hypothesis, this study utilized external datasets concerning individuals with a high BMI and AP to corroborate the findings. The results were promising, as the expression patterns of *HADH* genes aligned with the research findings in both datasets. At this juncture, there are sufficient grounds to consider *HADH* as a potential biomarker with diagnostic value in the development of AP in patients with a BMI >30.

## Advantages and limitations

This study integrated MR with bioinformatics data analysis to explore the crucial role of *HADH* in patients with AP and a BMI over 30, particularly regarding the immune response. It can facilitate more comprehensive and in-depth genetic research, expedite biological discovery, and enhance personalized medical outcomes for this patient population, thereby laying a theoretical foundation for personalized precision treatment. It is important to acknowledge that these findings are primarily based on computational analyses and existing data. Further validation through animal and cell experiments is imperative to confirm and extend these observations. Animal models, such as rodent models with genetic modifications related to *HADH* expression or activity, can offer a more direct means of elucidating the causative relationship between *HADH* and AP in patients with a BMI >30. By manipulating *HADH* expression levels or activity in vivo, this study revealed the resulting effects on pancreatic function, inflammation, and immune responses. These experiments provide a deeper understanding of the mechanistic interactions and pathways involved. Furthermore, this study only conducted corresponding analyses on the existing dataset, and the specific role of *HADH* in disease progression and outcomes still needs further research.

## Conclusion

In this study, a comprehensive approach was employed, leveraging MR, bioinformatics methods, and various machine learning algorithms to pinpoint *HADH* as a potential biomarker for AP in patients with a BMI >30, with a focus on lipid metabolism. These findings can empower clinicians to customize treatment strategies according to the diverse genetic profiles of BMI in patients with AP. Moreover, these findings pave the way for the development of medications specifically targeting *HADH* specifically to improve treatment efficacy and reduce side effects in patients with AP and a BMI exceeding 30.

### Supplementary Information

**Supplementary Material 1.** 

**Supplementary Material 2.** 

**Supplementary Material 3.** 

**Supplementary Material 4.** 

**Supplementary Material 5.** 

**Supplementary Material 6.** 

## Data Availability

The GWAS datasets analyzed in this study can be accessed from the IEU database (IEU OpenGWAS project (mrcieu.ac.uk) and ukb-a-248, ukb-b-19953, ukb-b-2303, and ukb-b-19388). The transcriptomic datasets analyzed in the present study are available in the [GEO] repository [https://www.ncbi.nlm.nih.gov/geo/and GSE151839, GSE44000, GSE194331, GSE109227, GSE166047].
