# Supplementary figures and images for: Investigating potential biomarkers of acute pancreatitis in patients with a BMI>30 using Mendelian randomization and transcriptomic analysis
Source: Lipids Health Dis. 2024 Apr 22;23:119. doi: 10.1186/s12944-024-02102-3 (PMC11034057; doi:10.1186/s12944-024-02102-3)

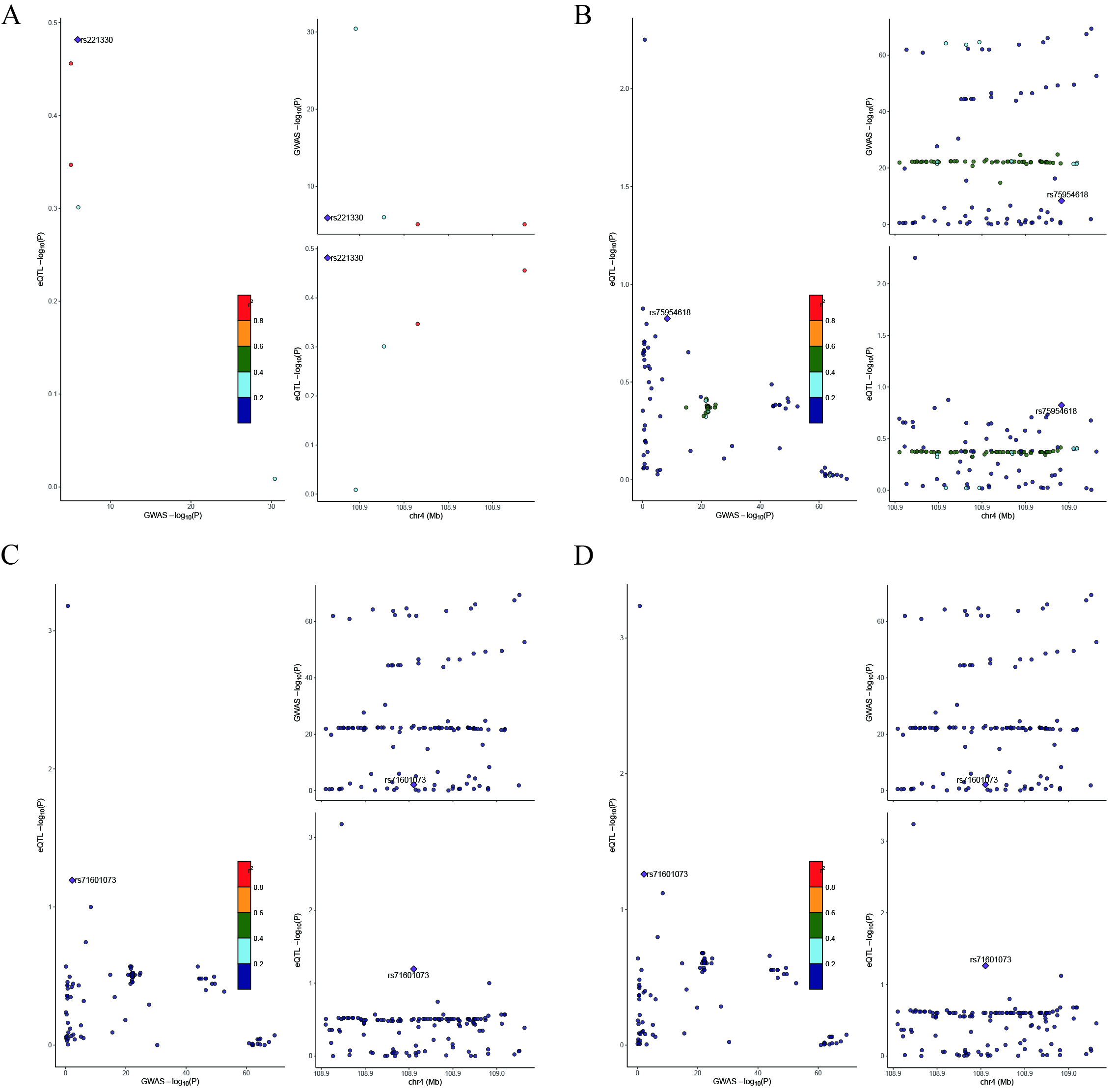

Supplement: Supplementary file 1 — Supplementary Material 1. [file 12944_2024_2102_MOESM1_ESM.tif]

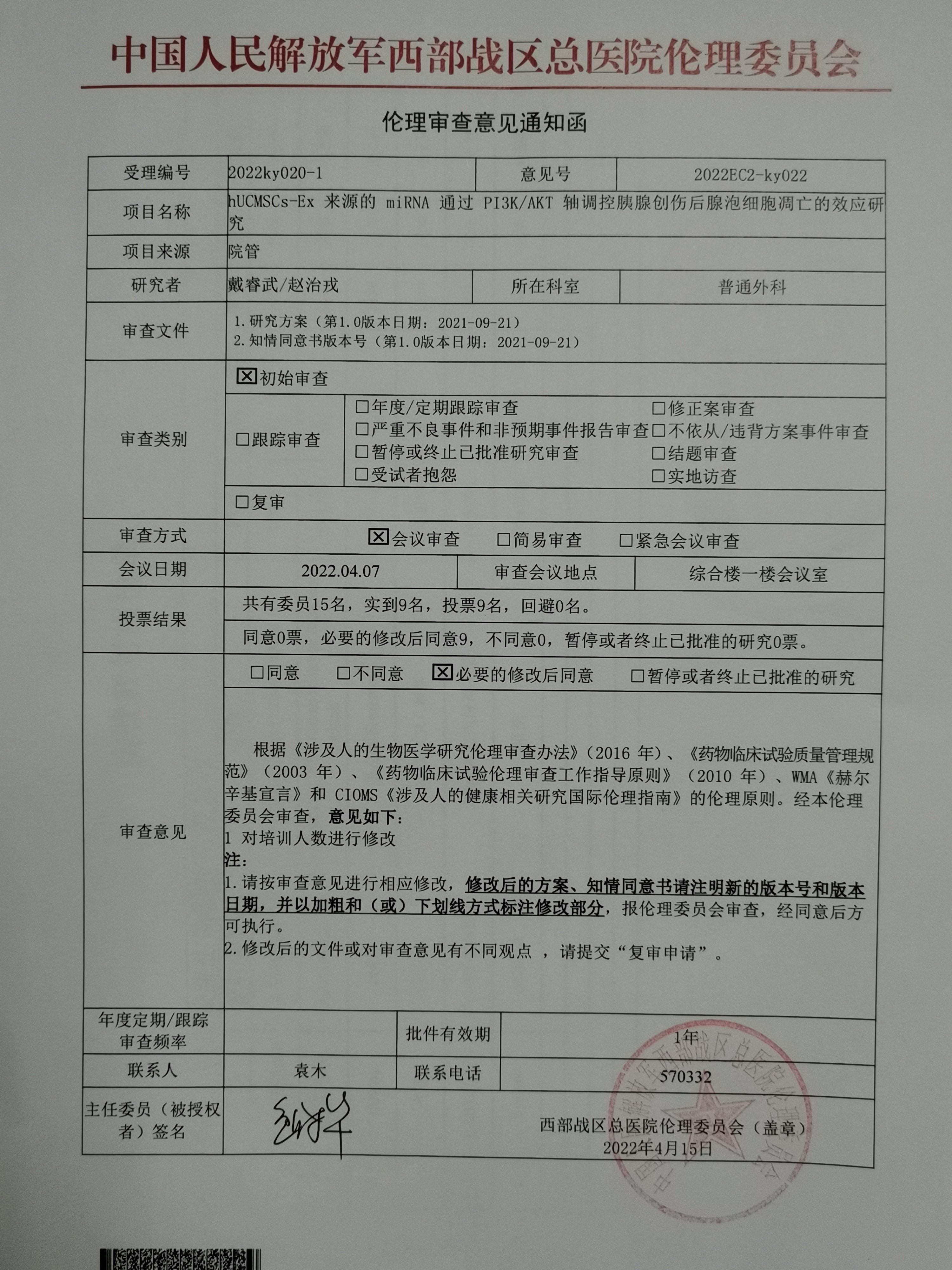

Supplement: Supplementary file 2 — Supplementary Material 2. [file 12944_2024_2102_MOESM2_ESM.jpg]

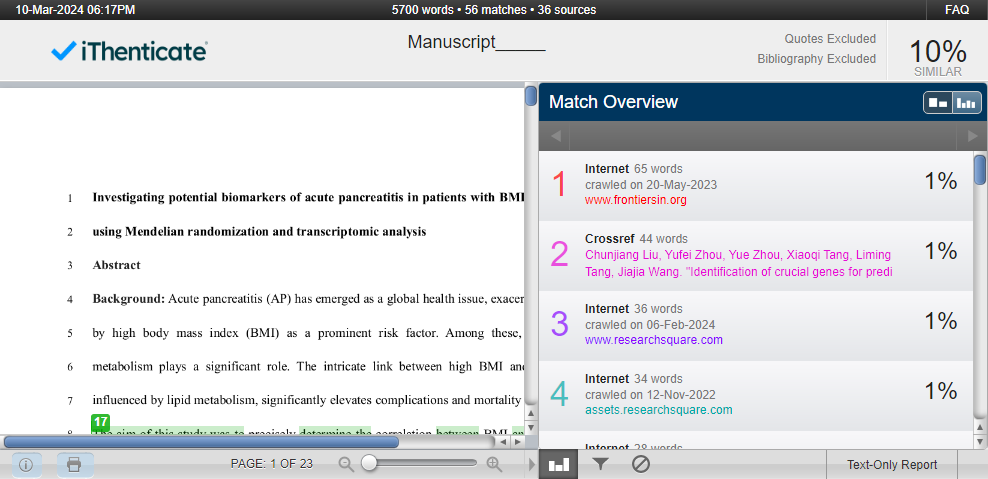

Supplement: Supplementary file 6 — Supplementary Material 6. [file 12944_2024_2102_MOESM6_ESM.png]
