# Supplementary material for: Investigating potential biomarkers of acute pancreatitis in patients with a BMI>30 using Mendelian randomization and transcriptomic analysis
Source: Lipids Health Dis. 2024 Apr 22;23:119. doi: 10.1186/s12944-024-02102-3 (PMC11034057; doi:10.1186/s12944-024-02102-3)
Supplement: Supplementary file 3 — Supplementary Material 3. [file 12944_2024_2102_MOESM3_ESM.docx]

Table S2

q-PCR primer sequences

| **Gene** | **Primer sequences** |
| --- | --- |
| HADH | F1:5'-ATGGCGTTCGTGACCAGGCA-3' |
|  | R1:5'-TGAAGCTTGAACGCTGCAAA-3' |
|  | F2:5'-CATCTTCCAGGTTTCTCCAC-3' |
|  | R2:5'-GGTTTCTCCACAAGCTTCAT-3' |
|  | F3:5'-AGGTTTGGTGTTTGCTGGCA-3' |
|  | R3:5'-TTGCTGGCAAACTGTGATCT-3' |
