# Supplementary material for: Investigating potential biomarkers of acute pancreatitis in patients with a BMI>30 using Mendelian randomization and transcriptomic analysis
Source: Lipids Health Dis. 2024 Apr 22;23:119. doi: 10.1186/s12944-024-02102-3 (PMC11034057; doi:10.1186/s12944-024-02102-3)
Supplement: Supplementary file 4 — Supplementary Material 4. [file 12944_2024_2102_MOESM4_ESM.docx]

Table S1

MR estimates of causal effect of 3 BMI IDs on acute pancreatitis.

| exposure | Outcome | Method | NO.SNP | Beta | SE | OR | CI | P-value |
| --- | --- | --- | --- | --- | --- | --- | --- | --- |
| ukb-a-248 | ukb−b−19388 | MR Egger | 176 | 0.0005 | 0.0014 | 1.0005 | 0.9978-1.0032 | 0.7166 |
|  |  | Weighted median | 176 | 0.0018 | 0.0008 | 1.0018 | 1.0002-1.0034 | <0.05 * |
|  |  | Inverse variance weighted | 176 | 0.0020 | 0.0004 | 1.0020 | 1.0012-1.0029 | <0.05 * |
|  |  | Simple mode | 176 | 0.0012 | 0.0019 | 1.0012 | 0.9975-1.0049 | 0.5307 |
|  |  | Weighted mode | 176 | 0.0012 | 0.0012 | 1.0012 | 0.9989-1.0035 | 0.3011 |
| ukb-b-2303 | ukb−b−19388 | MR Egger | 241 | 0.0019 | 0.0013 | 1.0019 | 0.9993-1.0046 | 0.1442 |
|  |  | Weighted median | 241 | 0.0019 | 0.0008 | 1.0019 | 1.0003-1.0035 | <0.05 * |
|  |  | Inverse variance weighted | 241 | 0.0020 | 0.0004 | 1.0020 | 1.0011-1.0028 | <0.05 * |
|  |  | Simple mode | 241 | 0.0018 | 0.0019 | 1.0018 | 0.9981-1.0056 | 0.3472 |
|  |  | Weighted mode | 241 | 0.0018 | 0.0013 | 1.0018 | 0.9993-1.0043 | 0.1573 |
| ukb-b-19953 | ukb−b−19388 | MR Egger | 240 | 0.0016 | 0.0013 | 1.0016 | 0.9990-1.0042 | 0.2374 |
|  |  | Weighted median | 240 | 0.0019 | 0.0008 | 1.0019 | 1.0003-1.0036 | <0.05 * |
|  |  | Inverse variance weighted | 240 | 0.0022 | 0.0004 | 1.0022 | 1.0013-1.0030 | <0.05 * |
|  |  | Simple mode | 240 | 0.0023 | 0.0019 | 1.0023 | 0.9985-1.0061 | 0.2458 |
|  |  | Weighted mode | 240 | 0.0020 | 0.0012 | 1.0020 | 0.9997-1.0044 | 0.0890 |
