# Supplementary material for: Investigating potential biomarkers of acute pancreatitis in patients with a BMI>30 using Mendelian randomization and transcriptomic analysis
Source: Lipids Health Dis. 2024 Apr 22;23:119. doi: 10.1186/s12944-024-02102-3 (PMC11034057; doi:10.1186/s12944-024-02102-3)
Supplement: Supplementary file 5 — Supplementary Material 5. [file 12944_2024_2102_MOESM5_ESM.docx]

Table S3

Body weight, pancreatic amylase and lipase in obese mice

| **Number** | **Weight(g)** | **Treatment** | **AMY（u/L）** | **LIP（u/L）** |
| --- | --- | --- | --- | --- |
| ① | 36.2 | physiological saline | 707 | 42.9 |
| ② | 32.5 | physiological saline | 1356 | 29.8 |
| ③ | 32.4 | physiological saline | 4095 | 25.5 |
| ④ | 32.9 | Taurocholic acid Sodium Salt | 155213 | 2499.7 |
| ⑤ | 33.8 | Taurocholic acid Sodium Salt | 222676 | 7533 |
| ⑥ | 32.4 | Taurocholic acid Sodium Salt | 28936 | 810.5 |
